# A phenome-wide association study to discover pleiotropic effects of *PCSK9*, *APOB*, and *LDLR*

**DOI:** 10.1038/s41525-019-0078-7

**Published:** 2019-02-11

**Authors:** Maya S. Safarova, Benjamin A. Satterfield, Xiao Fan, Erin E. Austin, Zhan Ye, Lisa Bastarache, Neil Zheng, Marylyn D. Ritchie, Kenneth M. Borthwick, Marc S. Williams, Eric B. Larson, Aaron Scrol, Gail P. Jarvik, David R. Crosslin, Kathleen Leppig, Laura J. Rasmussen-Torvik, Sarah A. Pendergrass, Amy C. Sturm, Bahram Namjou, Amy Sanghavi Shah, Robert J. Carroll, Wendy K. Chung, Wei-Qi Wei, QiPing Feng, C. Michael Stein, Dan M. Roden, Teri A. Manolio, Daniel J. Schaid, Joshua C. Denny, Scott J. Hebbring, Mariza de Andrade, Iftikhar J. Kullo

**Affiliations:** 1 Department of Cardiovascular Medicine, Mayo Clinic, Rochester, MN 55905 USA; 2 Biomedical Informatics Research Center, Marshfield Clinic Research Foundation, Marshfield, WI 54449 USA; 3 Department of Biomedical Informatics, Vanderbilt University, Nashville, TN 37235 USA; 4 Department of Genetics, University of Pennsylvania, Philadelphia, PA 19111 USA; 5 Department of Biomedical and Translational Informatics, Geisinger, Danville, PA 17821 USA; 6 Genomic Medicine Institute, Geisinger, Danville, PA 17822 USA; 7 Group Health Research Institute, Seattle, WA 98101 USA; 8 Department of Medicine (Medical Genetics), University of Washington Medical Center, Seattle, WA 98195 USA; 9 Department of Genome Sciences, University of Washington, Seattle, WA 98195 USA; 10 Genetic Services, Kaiser Permanente of Washington, Seattle, WA 98122 USA; 11 Department of Preventive Medicine, Northwestern University Feinberg School of Medicine, Chicago, IL 60611 USA; 12 Center for Autoimmune Genomics and Etiology, Cincinnati Children’s Hospital Medical Center, and Department of Pediatrics, University of Cincinnati, College of Medicine, Cincinnati, OH 45229 USA; 13 Division of Endocrinology, Cincinnati Children’s Hospital Medical Center and University of Cincinnati, Cincinnati, OH 45229 USA; 14 Department of Pediatrics, Columbia University, New York, NY 10032 USA; 15 Department of Medicine, Columbia University, New York, NY 10032 USA; 16 Division of Clinical Pharmacology, Department of Medicine, Vanderbilt University, Nashville, TN 37232 USA; 17 Department of Medicine, Vanderbilt University, Nashville, TN 37232 USA; 18 Division of Genomic Medicine, National Human Genome Research Institute, Bethesda, MD 20892 USA; 19 Department of Health Sciences Research, Mayo Clinic, Rochester, MN 55905 USA; 20 Center for Human Genetics, Marshfield Clinic Research Foundation, Marshfield, WI 54449 USA

## Abstract

We conducted an electronic health record (EHR)-based phenome-wide association study (PheWAS) to discover pleiotropic effects of variants in three lipoprotein metabolism genes *PCSK9*, *APOB*, and *LDLR*. Using high-density genotype data, we tested the associations of variants in the three genes with 1232 EHR-derived binary phecodes in 51,700 European-ancestry (EA) individuals and 585 phecodes in 10,276 African-ancestry (AA) individuals; 457 *PCSK9*, 730 *APOB*, and 720 *LDLR* variants were filtered by imputation quality (*r*^2^ > 0.4), minor allele frequency (>1%), linkage disequilibrium (*r*^2^ < 0.3), and association with LDL-C levels, yielding a set of two *PCSK9*, three *APOB*, and five *LDLR* variants in EA but no variants in AA. Cases and controls were defined for each phecode using the PheWAS package in R. Logistic regression assuming an additive genetic model was used with adjustment for age, sex, and the first two principal components. Significant associations were tested in additional cohorts from Vanderbilt University (*n* = 29,713), the Marshfield Clinic Personalized Medicine Research Project (*n* = 9562), and UK Biobank (*n* = 408,455). We identified one *PCSK9*, two *APOB*, and two *LDLR* variants significantly associated with an examined phecode. Only one of the variants was associated with a non-lipid disease phecode, (“myopia”) but this association was not significant in the replication cohorts. In this large-scale PheWAS we did not find LDL-C-related variants in *PCSK9*, *APOB*, and *LDLR* to be associated with non-lipid-related phenotypes including diabetes, neurocognitive disorders, or cataracts.

## Introduction

Genetic pleiotropy is widespread; ~5% of common variants and ~17% of genomic regions are associated with more than one phenotype.^1^ Genes implicated in lipoprotein metabolism are no exception and have been reported to be associated with type 2 diabetes.^2–5^ The National Human Genome Research Institute-European Bioinformatics Institute (NHGRI-EBI) Genome-wide Association Study (GWAS) catalog^4^ lists additional possible associations of variants near these genes with diverse diseases including Wilms’ tumor, allergic rhinitis, and bipolar disorder among others. Drugs specifically targeting genes or gene products involved in lipoprotein metabolism may therefore have unintended effects.^6,7^ Pathogenic variants in proprotein convertase subtilisin/kexin type 9 (*PCSK9*), apolipoprotein B (*APOB*), and low-density lipoprotein receptor (*LDLR*) can lead to familial hypercholesterolemia (FH). PCSK9 influences LDLR density on the hepatocyte surface and thereby low-density lipoprotein-cholesterol (LDL-C) levels through LDLR recycling.^8^ The gene product of *APOB* is found on LDL particles and is the ligand for LDLR.^9^

Recent reports demonstrate links between *LDLR* variants that lead to FH and decreased risk of diabetes.^2^ Conversely, statin therapy, which increases LDLR expression, is associated with risk of developing diabetes.^10^ Increased risk of diabetes was noted in carriers of the LDL-C lowering variant in *LDLR*, rs6511720.^11^ Monoclonal antibodies targeting PCSK9, and APOB antisense inhibitors are effective in lowering LDL-C levels and appear to lower the risk of atherosclerotic cardiovascular disease (ASCVD) events.^12–14^ The drugs have been approved for clinical use, however long-term safety data are lacking. In particular, several studies suggest that these drugs may increase risk of diabetes,^11,15,16^ neurocognitive impairment,^17–21^ and cataracts,^22^ although to date such associations have not been observed in prospective randomized control trials. The current study attempted to identify pleiotropic effects of variants in *PCSK9*, *APOB*, and *LDLR* that influence LDL-C levels with a particular focus on associations with diabetes, neurocognitive impairment, and cataracts given the concern raised in prior reports.

We conducted a comprehensive agnostic investigation of associations of *PCSK9*, *APOB*, and *LDLR* with non-lipid phenotypes on a phenome-wide scale to complement previous Mendelian randomization and post hoc analyses that raised concern of putative adverse associations. The phenome-wide association study (PheWAS) approach starts with genetic variants or genes of interest and then a large number of phenotypes are tested for association. Such an approach has revealed numerous previously unreported genotype–phenotype associations^23,24^ and provided insights into evolutionary genetics^25^ and drug repositioning.^26^ We attempted to extend on prior studies by including individuals of diverse ethnic backgrounds given the known differences in lipid levels by race/ethnicity^27–30^ and by the use of real-world patient electronic health record (EHR) data.

We leveraged high-density genotyping data linked to EHR-derived phenotypes from the electronic MEdical Records and GEnomics (eMERGE) Network^31,32^ to conduct a PheWAS to test the association of variants in *PCSK9*, *APOB*, and *LDLR* with non-lipid phenotypes, including diabetes, neurocognitive disorders, and cataracts. Associations were validated by conducting a cross validation in the eMERGE discovery cohort. Replication of significant *PCSK9*-trait, *APOB*-trait, and *LDLR*-trait associations was pursued in three independent cohorts: the Vanderbilt DNA biobank (BioVU) comprising individuals of European-ancestry (EA) and African-ancestry (AA), the Marshfield Personalized Medicine Research Project (PMRP), and the UK Biobank^33^ both comprised of EA individuals.

## Results

### Discovery cohort study population

Clinical characteristics of study participants from the discovery and three replication cohorts are shown in Table 1. Of the 83,985 individuals from the 12 eMERGE sites (Supplementary Table 1), 51,700 EA individuals (mean age 58 ± 16 years, 54% female) and 10,276 AA individuals (mean age 51 ± 16 years, 67% female) passed our quality control filters and had high-density genotyping data with imputed *PCSK9*, *APOB*, and *LDLR* variants, linked to the EHR.

**Table 1:** Clinical characteristics of study participants

Variable	Discovery Cohort (eMERGE Network) *N* = 62,210		Replication Cohort 1 (Marshfield PMRP) *N* = 9562	Replication Cohort 2 (BioVU) *N* = 29,713		Replication Cohort 3 (UK Biobank) *N* = 408,455
Race	EA	AA	EA	EA	AA	EA
*n*	51,700	10,276	9562	26,582	3131	408,455
Mean age years	58	51	62	62	61	57
Female (%)	54	67	62	58	52	54

*AA* African-ancestry; *BioVU* Vanderbilt DNA biobank; *EA* European-ancestry; *eMERGE* electronic MEdical Records and GEnomics Network; *PMRP* Marshfield Clinic Personalized Medicine Research Project

### Selection of variants

**Fig. 1:**
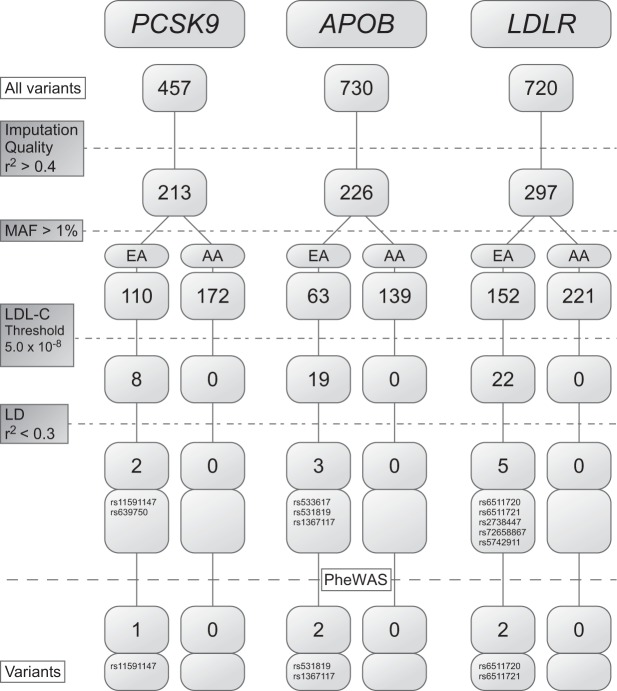
Selection of variants in the discovery cohort for the primary analysis. Collectively, individuals in the discovery cohort contained the number of variants shown for *PCSK9*, *APOB*, and *LDLR*. These variants were passed through various quality control filters and other selection measures including imputation quality (*r*^2^ > 0.4), minor allele frequency (MAF) > 1%, LDL-C association at the given thresholds for EA and AA, and linkage disequilibrium (*r*^2^ < 0.3). The variants passing these filters were used in the primary analysis. The rsID for each variant is shown

Collectively, individuals in the discovery set had 457 *PCSK9*, 730 *APOB*, and 720 *LDLR* variants. After applying quality control filters and other selection criteria including association with LDL-C, for the primary analysis, two *PCSK9*, three *APOB*, and five *LDLR* variants remained for PheWAS analysis in the EA cohort, but no variants remained for PheWAS analysis for the AA cohort (Fig. 1 and Table 2). Eight of these 10 variants had been tested in the Global Lipids Genetics Consortium (http://lipidgenetics.org/) and found to be significantly associated with LDL-C (Table 2).

**Table 2:** Variants that passed quality control filters in the primary analysis compared with the Global Lipids Genetics Consortium

Gene	Chr	Position^a^	rsID	Ref	Alt	Annotation	eMERGE cohort			GLGC metabochip		
							MAF EA (%)	Beta	*p-*value LDL-C	MAF in 1kGP (%)	Beta^b^	*p-*value
*PCSK9*	1	55505647	rs11591147	G	T	issense	1.4	−12.97	1.3 × 10^−27^	1.7	−0.50	1.6 × 10^−142^
		55519015	rs639750	T	G	Intron	32.7	−1.82	1.0 × 10^−9^	–	–	–
*APOB*	2	21233972	rs533617	T	C	Missense	3.8	−4.40	1.3 × 10^−9^	4.9	−0.14	1.7 × 10^−27^
		21263639	rs531819	G	T	Intron	15.5	−4.07	2.6 × 10^−26^	19.1	−0.12	1.3 × 10^−57^
		21263900	rs1367117	G	A	Missense	31.6	3.52	6.4 × 10^−32^	71.2	−0.11	1.4 × 10^−75^
*LDLR*	19	11202306	rs6511720	G	T	Regulatory intron	11.4	−5.79	4.2 × 10^−39^	9.8	−0.23	2.8 × 10^−151^
		11206575	rs6511721	A	G	Retained intron	48.3	1.73	5.6 × 10^−10^	48.8	−0.06	1.5 × 10^−29^
		11227480	rs2738447	C	A	Nonsense mediated decay	41.5	−1.67	4.0 × 10^−9^	42.9	−0.05	8.4 × 10^−13^
		11231203	rs72658867	G	A	Splice regions	1.1	−10.20	2.8 × 10^−14^	–	–	–
		11243445	rs5742911	A	G	3′ UTR	30.7	−1.79	3.7 × 10^−9^	26.8	−0.06	5.3 × 10^−24^

Selection criteria: Imputation quality *r*^2^ > 0.4; MAF > 1%; LCL-C association (threshold of 5.0 × 10^−8^); LD *r*^2^ < 0.3

*GLGC* Global Lipids Genetics Consortium, *Chr* chromosome number, *Ref* reference allele, *Alt* alternate allele, *MAF* minor allele frequency, *LDL-C* low-density lipoprotein cholesterol, *1kGP* 1000 Genomes program

^a^Position in human genome assembly hg19

^b^The difference in Beta between eMERGE and GLGC is primarily due to differences in units of measurements. eMERGE used mg/dL while GLGC used mmol/L

To determine whether variants not associated with LDL-C levels in the three genes were associated with other phenotypes, a secondary analysis was performed with a similar selection process in the discovery cohort that included “missense” variants not associated with LDL-C. This yielded four *PCSK9* (three in EA cohort, four in AA cohort), 15 *APOB* (5 in EA cohort, 12 in AA cohort), and one *LDLR* (one in both the EA and AA cohorts) variants suitable for PheWAS analysis (Supplementary Figure 1; Supplementary Table 2).

### Selection of phecodes

**Fig. 2:**
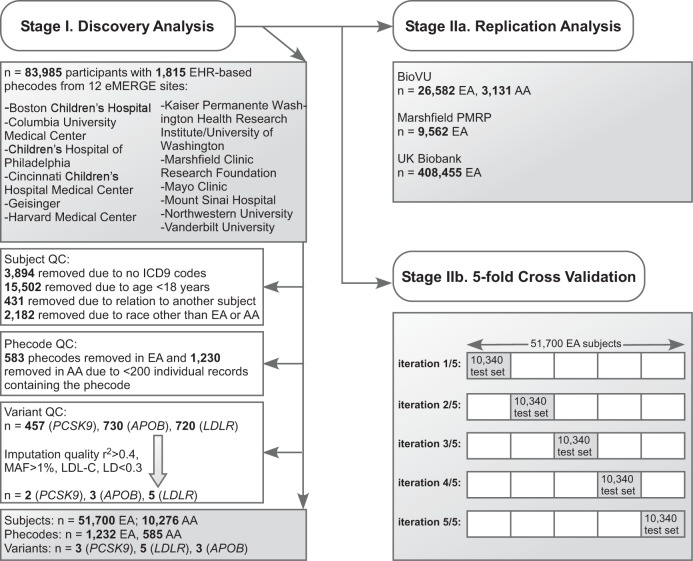
Study outline for primary analysis. AA African-ancestry, EA European-ancestry, EHR electronic health record, eMERGE electronic MEdical Records and GEnomics Network, LD linkage disequilibrium, PMRP Personalized Medicine Research Project, QC quality control

Of the 1815 available phenotypes, 1232 and 585 passed quality control filters for the EA and AA cohorts, respectively (Supplementary Data 1). Phecodes representing diabetes, neurocognitive disorders, and cataracts are listed in Supplementary Tables 3–5, respectively. A summary of the selection strategy for participants, variants, and phecodes, as well as the replication analysis and five-fold cross validation is shown in Fig. 2.

### PheWAS results

In the discovery cohort, the PheWAS identified one *PCSK9*, two *APOB*, and two *LDLR* variants in the EA sample that were significantly associated (*p* < 5.8 × 10^−5^) with an examined phecode (Fig. 1 and Table 3). Only one of the variants, the *LDLR* variant rs6511720, was associated with a non-lipid/non-ASCVD phecode, that being “myopia.” These five variants underwent additional analyses described below. Several of the variants trended towards association with ischemic heart disease, with the strongest association seen for rs639750 in *PCSK9* (*p* = 0.0065, OR 0.96).

**Table 3:** Significant associations in the discovery and replication cohorts

Phecode	Description	Variant	MAF (%)	eMERGE discovery cohort					eMERGE 5-fold cross validation	Marshfield replication cohort			Vanderbilt replication cohort			UK Biobank
				Cases	Controls	*p* value^a^	Odds ratio^b^	95% CI	*p* value^b^	Cases	Controls	*p* value^a^	Cases	Controls	*p* value^a^	*p* value^a^
*Lipid-related phecode associations*																
272	Disorders of lipid metabolism	rs11591147	1.4	25,298	17,205	**3.16** **×** **10**^−**12**^	0.64	0.51–0.76	**1.2** **×** **10**^−**10**^	8291	1796	**8.2** **×** **10**^−**5**^	9076	14,560	**3.5** **×** **10**^−**3**^	**2.1** **×** **10**^−**28**^
		rs531819	15.5	25,298	17,205	**2.30** **×** **10**^−**9**^	0.88	0.84–0.92	**1.4** **×** **10**^−**8**^							
		rs1367117	31.5	25,298	17,205	**2.55** **×** **10**^−**5**^	1.07	1.04–1.10	5.4 × 10^−4^				9314	18,219	**6.7** **×** **10**^−**4**^	**8.8** **×** **10**^−**22**^
		rs6511720	11.4	25,298	17,205	**2.78** **×** **10**^−**15**^	0.83	0.78–0.87	**7.0** **×** **10**^−**15**^	7852	1710	**5.1** **×** **10**^−**3**^	9346	18,219	**1.3** **×** **10**^−**4**^	**2.0** **×** **10**^−**48**^
		rs6511721	48.3	25,298	17,205	**1.73** **×** **10**^−**5**^	1.07	1.04–1.10	4.0 × 10^−4^							**2.5** **×** **10**^−**16**^
272.1	Hyperlipidemia	rs11591147	1.4	25,168	17,205	**2.84** **×** **10**^−**12**^	0.64	0.51–0.76	**9.6** **×** **10**^−**11**^	7666	1796	**1.8** **×** **10**^−**5**^	9050	14,560	**3.9** **×** **10**^−**3**^	
		rs531819	15.5	25,168	17,205	**1.35** **×** **10**^−**9**^	0.88	0.84–0.92	**8.4** **×** **10**^−**9**^							
		rs1367117	31.5	25,168	17,205	**1.41** **×** **10**^−**5**^	1.07	1.04–1.11	3.6 × 10^−4^				9346	18,219	**1.0** **×** **10**^−**3**^	**2.1** **×** **10**^−**93**^
		rs6511720	11.4	25,168	17,205	**3.27** **×** **10**^−**15**^	0.83	0.78–0.87	**9.8** **×** **10**^−**15**^	7259	1710	**3.1** **×** **10**^−**3**^	9314	18,219	**9.8** **×** **10**^−**5**^	**6.7** **×** **10**^−**124**^
		rs6511721	48.3	25,168	17,205	**1.91** **×** **10**^−**5**^	1.07	1.04–1.10	4.6 × 10^−4^							**8.5** **×** **10**^−**31**^
272.11	Hypercholesterolemia	rs11591147	1.5	11,753	17,205	**3.62** **×** **10**^−**10**^	0.60	0.44–0.76	**3.4** **×** **10**^−**8**^	5602	1796	**2.5** **×** **10**^−**5**^	3840	14,560	**8.0** **×** **10**^−**4**^	**6.3** **×** **10**^−**72**^
		rs531819	15.7	11,753	17,205	**6.49** **×** **10**^−**8**^	0.87	0.81–0.92	**1.2** **×** **10**^**−7**^							
		rs6511720	11.5	11,753	17,205	**5.33** **×** **10**^−**14**^	0.80	0.74–0.86	**6.1** **×** **10**^−**13**^	5316	1710	**1.4** **×** **10**^−**3**^	3953	18,219	**2.7** **×** **10**^−**3**^	
272.13	Mixed hyperlipidemia	rs6511720	12.0	4942	17,205	**3.90** **×** **10**^−**6**^	0.84	0.76–0.91	**7.1** **×** **10**^−**5**^	147	1710	3.6 × 10^−1^	4572	18,219	**8.5** **×** **10**^−**4**^	
*Non-lipid-related phecode associations*																
367.1	Myopia	rs6511720	11.4	4138	36,272	**1.76** **×** **10**^−**5**^	0.85	0.77–0.92	^c^8.8 × 10^−4^	3879	1868	4.5 × 10^−1^	823	27,142	3.5 × 10^−1^	4.6 × 10^−1^

ICD-9 codes were extracted from individual EHRs and converted to phecodes using the PheWAS R package

CI confidence interval, LDL-C low-density lipoprotein cholesterol, MAF minor allele frequency

^a^Bold values are statistically significant

^b^Odds ratio refers to the Alt allele

^c^Borderline significant, other variants in LD with this variant were significant

A secondary PheWAS analysis of additional missense variants not associated with LDL-C was performed. None of these variants were significantly associated with a phecode in the EA or AA cohorts; therefore, no further tests with these variants were performed.

Our analyses included EA and AA individuals. However, when we included the remaining 2182 non-EA/non-AA individuals (Supplementary Table 1) with the EA group, our inferences were similar.

Two low-frequency *PCSK9* variants, rs67608943 and rs28362286, have been associated with lower LDL-C levels in AA individuals. As no AA variants passed our selection criteria for PheWAS analysis, we performed an additional analysis with these two variants, but did not find these variants to have any significant associations.

### Myopia association

There were 16 *LDLR* variants in LD (*r*^2^ > 0.3) with rs6511720 that were also associated with myopia. Of these, rs2228671 had the strongest association with “myopia” but a weaker association with the lipid-related phecodes. Manhattan plots of phecode associations of the *LDLR* variants rs6511720 (Supplementary Figure 2a) and rs2228671 (Supplementary Figure 2b) highlight that these variants, although in LD, have varying strengths of association. Supplementary Figure 3 presents the strength of association with the phecode “hypercholesterolemia” or LDL-C levels, myopia, and myopia adjusted for the phecode “hypercholesterolemia” or LDL-C levels for the 16 variants in LD. The strength of association with myopia was attenuated but remained significant after adjustment for hypercholesterolemia or LDL-C levels. Based on LD the 16 variants associated with myopia could be placed into four groups (Supplementary Figure 3). Variants in the same group had an *r*^2^ > 0.98. The variant rs6511720 (blue), relatively distant from the remaining variants, had the strongest association with LDL-C level. rs2228671 (green) along with another nine variants in its group were most strongly associated with myopia.

When eMERGE consortium site was added as a covariate in the analysis, the signal for myopia was no longer significant, suggesting that one or a few sites were driving the association.

### Cross validation and replication

Using five-fold cross validation, most of the lipid-related phecode associations of the *PCSK9*, *APOB*, and *LDLR* variants remained significant (*p* < 4.1 × 10^−5^). The association between the *LDLR* variant rs6511720 and the phecode “myopia” was borderline significant (Table 3). Other variants in LD with rs6511720 also had borderline significant associations with the phecode “myopia.” When eMERGE consortium site was added as a covariate in the cross validation analysis, the signal for myopia was no longer significant, again, suggesting that one or a few sites were driving the association. All lipid-related phecode associations from the *PCSK9*, *APOB*, and *LDLR* variants were replicated in the Marshfield PMRP, BioVU and/or UK cohorts; however, the non-lipid association of rs6511720 with the phecode “myopia” was not confirmed in any of the replication cohorts (Table 3).

### Comparison to the GWAS catalog

We examined the NHGRI-EBI GWAS catalog^4^ for all reported variants within the boundaries of *PCSK9*, *APOB*, and *LDLR*. We found 27 variants (4 in *PCSK9*, 14 in *APOB*, and 9 in *LDLR*) with 86 reported associations. Six of these variants were protein-function altering, either missense or stop-gain. Two variants were not available in the eMERGE dataset; therefore, we tested the remaining 70 associations in the eMERGE dataset. From those 70, 28 had significant lipid associations and no significant pleiotropic effects (cross-phenotype associations) were present, including lack of association with “myopia.” Eight variants were not available in the UK Biobank dataset; therefore, we tested the remaining 55 associations in the UK Biobank. All of these were significant replicating previously reported associations with lipid levels, ischemic heart disease, and disorders of lipoprotein metabolism. There were no significant pleiotropic effects (including lack of association with “myopia”). A list of reported associations with the UK Biobank code descriptions and eMERGE phecode equivalent is presented in Supplementary Data 2.

### Power

We calculated power using the R package “powerMediation”. For logistic regression analyses with phecode as the binary outcome and genotypes as discrete predictors, power was calculated for each pair of variant and phecode, based on sample size, allele frequency for each variant, odds ratio (OR) and type I error *α* = 4.1 × 10^−5^. We had more than 80% power to detect 30% of associations in EA individuals. However power for individual variants was low (Supplementary Figure 4); for higher frequency variants, power for the phecodes “ischemic heart disease” and “type 2 diabetes”, was 0.175 and 0.143, respectively.

## Discussion

In a large PheWAS we confirmed the association of *PCSK9*, *APOB*, and *LDLR* with disorders of lipid metabolism (hypercholesterolemia) at the variant level. We found no evidence that variation in *PCSK9*, *APOB*, and *LDLR* is associated with diabetes or any non-lipid phenotypes including neurocognitive disorders or cataract. This includes the *PCSK9* variant rs11591147 and the *LDLR* variant rs6511720 for which prior studies have reported borderline significant associations with increased risk of diabetes.^11,34^ In the NHGRI-EBI GWAS catalog, no associations of *PCSK9*, *APOB*, or *LDLR* variants with diabetes, neurocognitive disorders, or cataract have been reported. Additionally, an examination of the UK Biobank all-by-all PheWAS browser (http://pheweb.sph.umich.edu) did not demonstrate pleiotropic effects for any tested variants in *PCSK9*, *APOB*, or *LDLR*.

In our discovery cohort we identified an association of several variants in *LDLR* with “myopia”, but none of these were confirmed in the replication cohorts and only the association between some *LDLR* variants including rs2228671 and “myopia” was present on five-fold cross validation. We were unable to find any physiological basis in the literature for an association between lipid level or lipid genes and myopia, and given the lack of replication, this could be a false positive association.

Long-term safety data on PCSK9 inhibitors are not available given the limited follow up of clinical trials that have been conducted so far.^35^ In particular, there is a theoretical concern for increased risk of diabetes, neurocognitive disorders, and cataracts. The U.S. Food and Drug Administration issued a directive to monitor for adverse neurocognitive events in patients treated with PCSK9 inhibitors,^36^ and ongoing pharmacovigilance programs are in place. In our analysis, we did not find a significant association between *PCSK9* variation and neurocognitive disorders apart from the borderline association with “myopia”.

In the NHGRI-EBI GWAS catalog^4^ common variants at the *PCSK9* and *APOB* loci were associated with non-lipid/non-ASCVD traits.^37–45^ Most of these variants were intergenic and were therefore excluded from our study which only included variants within the gene borders. Three variants (rs6006893, rs219553, and rs2495478) were intronic and therefore of uncertain functional significance. The association of variant rs2495478 with Wilms’ tumor was not replicated and the other two variants were not present in the eMERGE dataset to compare. The UK Biobank PheWAS browser also did not list any of these associations reported in the GWAS catalog (Supplementary Data 2). Therefore, we did not confirm the associations reported in the NHGRI-EBI GWAS catalog for variants available in our analyses.

Two recent studies reported differing results regarding the association between the LDL-C lowering variant rs11591147 and risk of diabetes.^11,34^ In a Mendelian randomization study *PCSK9* variants associated with low LDL-C levels (rs11583680, rs11591147, rs2479409, and rs11206510) modestly increased risk of diabetes (OR 1.29; 1.11–1.50).^15^ A meta-analysis encompassing 50,775 individuals with type 2 diabetes and 270,269 control subjects revealed an OR of 1.09 for rs11591147, a cholesterol-lowering variant^11^ matching an OR of 1.11 (1.04–1.19) for each 10 mg *PCSK9*-mediated decrease in LDL-C levels.^16^ Circulating PCSK9 levels are increased in patients with diabetes and metabolic syndrome.^46^ On the other hand, a recent report found no association between rs11591147 and markers of glucose homeostasis or diabetes^34^ and no evidence of increased risk of new-onset diabetes was found in a pooled analysis of 10 phase III trials of PCSK9 inhibitors with a follow-up period of 6–18 months.^47^ Additional studies and longer-term follow-up of PCSK9 inhibitors may be needed to confirm/refute an association with diabetes.

Individuals with FH have been reported to have decreased risk of diabetes and there are also links between the use of statins and an increased risk from diabetes. However, no studies have identified an association between specific *APOB* or *LDLR* variants and diabetes. We also did not find any association with specific variants in these genes with any of the 19 phecodes associated with diabetes. Of note, a recent GWAS report described that only a very small fraction of LDL-C lowering genetic variants (only 5 out of 113 variants from 90 distinct loci) were associated with type 2 diabetes.^48^ None of these were in *PCSK9*, *APOB*, or *LDLR*. However, a lack of pleiotropic effects in a subset of variants does not exclude the possibility of pleiotropic effects for other variants in the studied genes or in other ethnic backgrounds.

We evaluated the previously reported association between lipid-lowering drugs and the risk of cataracts^17,18^ but observed no significant signal for *PCSK9*, *APOB*, or *LDLR* and any of the six tested phecodes pertinent to cataracts. We did not find the loss-of-function rs11591147 (R46L) variant to be associated with hemorrhagic stroke, although low LDL-C levels on lipid-lowering drugs have been associated with the risk of intracerebral hemorrhage.^49^

While this manuscript was being reviewed, two sets of PheWAS results were published for *PCSK9* variants. In the first,^50^ a gene-centric score derived from four *PCKS9* variants (rs11583680, rs11591147, rs2479409, and rs11206510) that were associated with LDL-C in the Global Lipids Genetics Consortium (http://lipidgenetics.org/) was associated with myocardial infarction and type 2 diabetes. Associations for individual variants were not reported. The second of these studies^51^ examined only a single *PCSK9* variant, rs11591147, in 337,536 individuals of predominantly European ancestry in the UK Biobank and demonstrated it to be associated with hyperlipidemia and coronary heart disease, which is similar to our results which trended toward association with ischemic heart disease but not with type 2 diabetes. Neither of these studies found any associations for *PCSK9* variants with neurocognitive disorders and cataracts, nor did these examine variants in *APOB* or *LDLR*.

In summary, our primary analysis identified only one pleiotropic effect, “myopia” in the discovery cohort for *LDLR*, which remained borderline significant on five-fold cross validation and was not replicated in any of the three replication cohorts. A PheWAS for missense variants not associated with LDL-C also did not identify any pleiotropic effects. Lastly, we did not replicate the associations reported in the NHGRI-EBI GWAS catalog for *PCSK9*, *APOB*, and *LDLR* variants.

### Strengths and limitations

The present study included a larger sample size of AA individuals than previous PheWAS analyses. Also, in addition to correcting for multiple testing, we evaluated significant results in a large discovery cohort, three large independent replication cohorts, and conducted five-fold cross validation. Replication of the known associations with LDL-C^52^ in directions consistent with previous epidemiologic and genetic studies provided an internal validation of our PheWAS approach. Our primary analysis was restricted to only functional *PCSK9*, *APOB*, and *LDLR* variants but we did perform a secondary analysis including only “missense” mutations with similar results.

Several limitations are worth noting. First, EHRs are a repository of longitudinal data that capture phenotypes with varying resolution, thus their use for research may be subject to misclassification; some control subjects may have limited contact with the health care system possibly leading to misclassification in those individuals. Second, although the sample size of AA individuals was larger than previous studies, it was relatively small compared to the EA cohort and may not be sensitive in detecting pleiotropic associations. Given that genetic structure varies across populations of different ancestry backgrounds, there is a need to assess phenotype–genotype associations in diverse ethnic groups, including individuals of African, Asian, and Hispanic/Latino ancestry. Third, the phecodes in UK Biobank did not correspond exactly to the phecodes in the eMERGE cohort so best approximations had to be applied. Fourth, although the associations between the LDL-C-related variants and ischemic heart disease trended towards significance, these did not reach the Bonferroni threshold, highlighting that there could be pleiotropic associations that were simply below the threshold of detection in our dataset. Fifth, general limitations of the PheWAS approach that are not specific to our study include low power to detect weaker pleiotropic effects and inability to directly address potential off-target side effects of pharmacologic manipulation of the examined genes.

### Conclusion

In this large-scale PheWAS we did not find LDL-C associated or missense variants in *PCSK9*, *APOB*, and *LDLR* to be associated with non-lipid phenotypes; specifically no association was seen with neurocognitive disorders, diabetes, or cataracts. These data suggest a lack of major pleiotropic effects of the tested *PCSK9*, *APOB*, and *LDLR* variants.

## Methods

### Genotyping, quality control, and selection criteria

High-density genotype data were available for 83,985 participants of the eMERGE network. To unify the genotype data processed on 78 different chips from 12 contributing sites, each genotype array batch was imputed via the Michigan Imputation Server (MIS; https://imputationserver.sph.umich.edu/) and all imputed batches of data were combined into a unified dataset. The imputation was based on minimac3 algorithm^53^ and the genotype reference panel was from Haplotype Reference Consortium.^54^ All research activities were reviewed and approved by the Institutional Review Board (IRB) at each eMERGE site and all research subjects gave written informed consent.

Medications were extracted from prescription databases and/or clinic notes for each institution. Lipid lowering medications (LLMs) included: cerivastatin, rosuvastatin, simvastatin, fluvastatin, pravastatin, lovastatin, atorvastatin, and pitavastatin. For the majority (76.3%) of participants, we used median LDL-C levels prior to the use of any LLM. For the remaining 23.7% of participants with LDL-C levels while on LLM, the median LDL-C level was divided by 0.75 to impute LDL-C levels prior to initiating LLM^55^ assuming a 25% reduction in LDL-C on therapy. To assess association with LDL-C, we used an additive genetic model with age, sex, LLM status, and the first two principal components as covariants.

For the primary analysis we tested variants meeting the following criteria: within the *PCSK9*, *APOB*, or *LDLR* gene boundary (using NCBI gene reference; *PCSK9*, chromosome 1: 55505149–55530526; *APOB*, chromosome 2: 21224301–21266945, *LDLR*, chromosome 19: 11200037–11244506), minor allele frequency (MAF) > 1%, high imputation quality (*r*^2^ > 0.4), associated with LDL-C level, and not in linkage disequilibrium (*r*^2^ < 0.3). For a group of variants in LD, we picked the one with strongest association with LDL-C. The standard GWAS genome-wide threshold of significance of <5.0 × 10^−8^ was used for both the EA and AA cohorts to determine association with LDL-C.

For the secondary analysis we tested all variants meeting the following criteria: within the *PCSK9*, *APOB*, or *LDLR* gene boundary, MAF > 1%, missense variants that were not associated with LDL-C level, high imputation quality (*r*^2^ > 0.4), and not in linkage disequilibrium (*r*^2^ < 0.3). SeattleSeq (http://snp.gs.washington.edu/SeattleSeqAnnotation138/) was used to annotate variant function including identifying missense mutations.

We randomly removed one from each related pair of participants (first degree of relatives) using identity-by-descent (IBD) measures $\hat p \ge 0.5$.^56^ We performed principal component analysis in the eMERGE cohort and 2504 samples from the 1000 Genomes Project phase 3^57^ to infer genetic ancestry. We also stratified analyses for AA individuals and EA individuals. We restricted our analyses to adults (age > 18 years). If any participant had only one instance or encounter for any of the component ICD codes, he/she was excluded from the analysis of the corresponding phecode.

### Phenotyping

We converted International Classification of Diseases, Ninth Revision (ICD-9) codes from EHRs to 1815 phecodes^58^ using PheWAS package.^59^ A ‘case’ for a given phecode was defined as having a minimum of two ICD-9 codes on different dates. Controls did not have any related phecodes according to the exclusion criteria embedded in the PheWAS package. To retain statistical power, we only analyzed phecodes with ≥200 cases.^60^

### Statistical analysis

Associations between single variants in *PCSK9*, *APOB*, and *LDLR* and individual phecodes were performed in the eMERGE discovery cohort stratified by genetically inferred ancestry (AA and EA individuals) as described above. In an effort to include all participants regardless of ancestry, we performed an additional analysis where we grouped all non-AA ancestries with EA. Logistic regression assuming an additive genetic model was utilized with adjustment for median age at which ICD-9 codes were recorded, sex, and the first two principal components from our evaluation of genetic ancestry described above. A scree plot showed that the first two principal components captured 79% of the variates (Supplementary Figure 5M). A Bonferroni threshold of significance was defined as 0.05/(number of tested phecodes). PheWAS analyses were repeated with site added as a covariate.

### Myopia association

The discovery cohort contained 15 additional variants that were in LD (*r*^2^ > 0.3) with rs6511720 and tested against hypercholesterolemia code/LDL-C levels, myopia code and myopia code adjusted for hypercholesterolemia code/LDL-C levels.

### Cross validation

We used cross validation in the discovery cohort dataset for associated phenotypes. This methodology simulates tests on the independent test dataset and aims to prevent over-fitting.^61^ In cross validation, we partitioned at random a given dataset into five equally sized subsets/folds. Then, one of the subsets was used to detect association, and this was repeated four times so that each subset was used once to perform the test. We combined the results from the five tested folds together using Fisher’s method,^62^ which corresponds to performing tests on all samples. Cross-validation analysis was repeated with site included as a covariate.

### Replication

Significant variant-phecode associations were evaluated in three separate cohorts. The BioVU,^63^ Marshfield Clinic Biobank,^64^ and the UK Biobank^33^ included 29,713, 9562, and 408,455 participants, respectively. To avoid overlap between the discovery and the replication cohorts, the BioVU and Marshfield Clinic Biobank replication cohorts only included individuals who were not eMERGE participants.

All UK Biobank participants for whom PheWAS results were available were included in the number above. Replication in the available datasets was defined as *p*-value < 0.05/number of replicated variants.

### Testing association reported in the GWAS catalog

We tested whether the previously reported associations for variants in the three lipid metabolism genes were present in the eMERGE dataset and UK Biobank. We collected all the variants within the boundaries of the three genes that were listed in the National Human Genome Research Institute- European Bioinformatics Institute (NHGRI-EBI) GWAS catalog.^4^ A physician mapped the phenotypes from the GWAS catalog to the closest codes used in the PheWAS package and UK Biobank. Mapping is available in Supplementary Data 2. Unmapped phenotypes were not further analyzed. We tested the association pairs in the eMERGE dataset and extracted the statistical values from the Gene ATLAS PheWAS website from UK Biobank. We used *p*-value 0.05 as the threshold for replication.

### Power calculation

Power for a given sample size, MAF, OR, and type I error = significance level = 0.05/# of tested phecodes (*α* = 4.1 × 10^−5^) was calculated for each variant-phecode pair.^65^ We summarized the power to detect associations in the EA dataset. Additionally, we calculated the post-hoc power for the phecode “ischemic heart disease” (by grouping all ICD 9 codes 411–414), type 2 diabetes, and the 10 tested genetic variants.

### Reporting summary

Further information on experimental design is available in the Nature Research Reporting Summary linked to this article.

## Supplementary information

Supplemental Information

Supplemental Data 1

Supplemental Data 2

Reporting Summary

## Data Availability

The datasets analyzed during the current study are available in the database of Genotypes and Phenotypes (dbGaP); dbGaP Study Accession: phs000888.v1.p1.

## Supplementary information

**Supplementary information** accompanies the paper on the *npj Genomic Medicine* website (10.1038/s41525-019-0078-7).
