# Supplementary material for: A phenome-wide association study to discover pleiotropic effects of PCSK9, APOB, and LDLR
Source: NPJ Genom Med. 2019 Feb 11;4:3. doi: 10.1038/s41525-019-0078-7 (PMC6370860; doi:10.1038/s41525-019-0078-7)
Supplement: Supplementary file 1 — Supplemental Information [file 41525_2019_78_MOESM1_ESM.pdf]

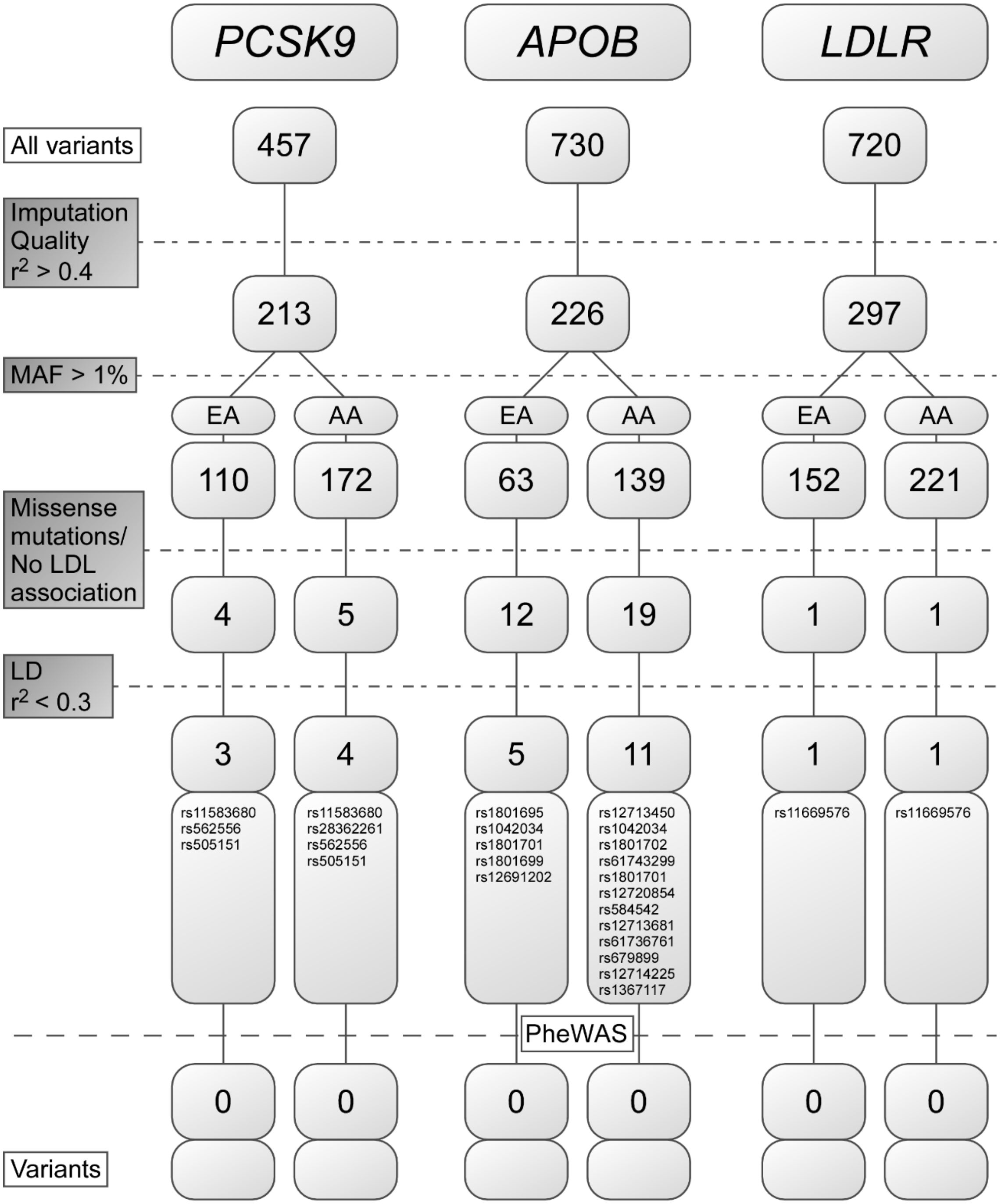

Supplementary Figure 1. Selection of variants in the discovery cohort for the secondary analysis. The discovery cohort contained the number of variants shown for *PCSK9*, *APOB*, and *LDLR*. These variants were passed through various quality control filters and other selection measures including imputation quality (r^2 >0.4), minor allele frequency (MAF) >1%, Missense mutations not associated with LDL-C, and linkage disequilibrium (r^2 <0.3). The variants passing these filters were used in the secondary analysis. The rsID for each variant is shown.

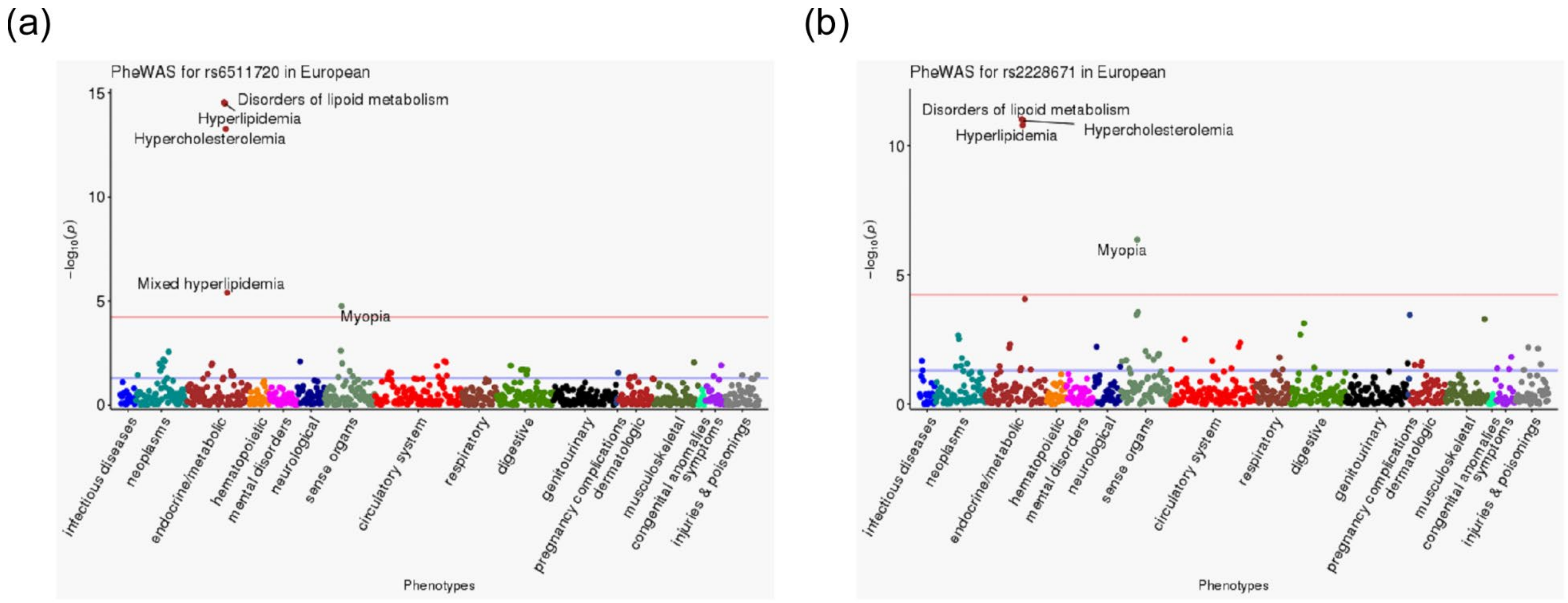

Supplementary Figure 2. Myopia association Manhattan plots. (a) Manhattan plot demonstrating phecode associations for *LDLR* variant rs6511720. **(b)** Manhattan plot demonstrating phecode associations for *LDLR* variant rs2228671.

(a)

SNP	rsID
19:11202306	rs6511720
19:11205975	rs8102912
19:11206729	rs17242381
19:11206969	rs17242395
19:11207102	rs1010679
19:11207777	rs117423069
19:11207982	rs67337506
19:11209576	rs17248769
19:11209629	rs73015033
19:11209722	rs73015034
19:11209764	rs74857287
19:11210254	rs17248776
19:11210314	rs17248783
19:11210912	rs2228671
19:11212470	rs12985460
19:11214533	rs2569559

LD	Blue	Green	Purple	Red
Blue		0.68	0.37	0.33
Green			0.42	0.50
Purple				0.87
Red				

(b)

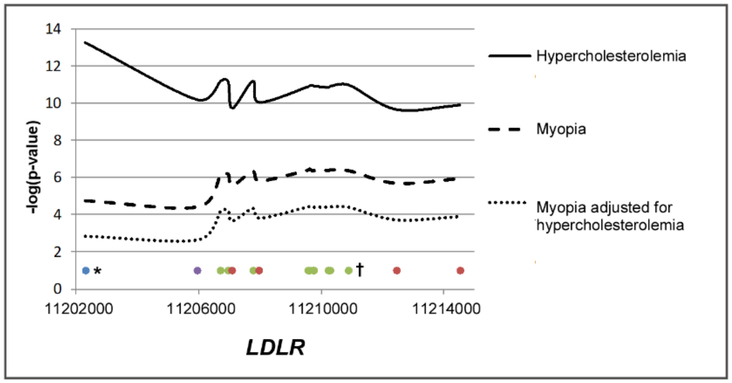

(c)

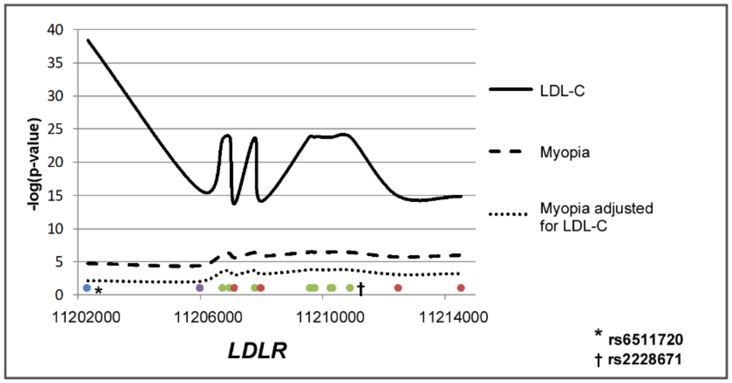

(d)

LD	rs6511720	rs8102912	rs17242381	rs17242395	rs1010679	rs117423069	rs67337506	rs17248769	rs73015033	rs73015034	rs74857287	rs17248776	rs17248783	rs2228671	rs12985460	rs2569559
rs6511720																
rs8102912	0.375803															
rs17242381	0.674679	0.42061														
rs17242395	0.674456	0.420697	0.998275													
rs1010679	0.332547	0.87103	0.505006	0.504867												
rs117423069	0.674749	0.42056	0.998276	0.997901	0.505373											
rs67337506	0.332657	0.867577	0.504787	0.504648	0.994102	0.505409										
rs17248769	0.668122	0.417806	0.989743	0.989958	0.501813	0.991164	0.502436									
rs73015033	0.668014	0.417711	0.989518	0.990032	0.501717	0.991239	0.50234	0.999774								
rs73015034	0.668283	0.417775	0.989667	0.989882	0.501805	0.991089	0.502428	0.999172	0.999097							
rs74857287	0.668055	0.417707	0.989444	0.989658	0.501715	0.990865	0.502253	0.999548	0.999322	0.999473						
rs17248776	0.66775	0.417581	0.989369	0.989584	0.501575	0.99079	0.502197	0.999624	0.999398	0.999247	0.999624					
rs17248783	0.667585	0.417738	0.989144	0.989359	0.501746	0.990565	0.502369	0.999398	0.999172	0.999021	0.999398	0.999774				
rs2228671	0.668321	0.417843	0.989668	0.989882	0.501872	0.991089	0.502495	0.998871	0.998645	0.999247	0.998871	0.999247	0.999021			
rs12985460	0.338231	0.84214	0.49836	0.498723	0.964129	0.499062	0.968812	0.504617	0.504521	0.50444	0.504688	0.504717	0.504889	0.504423		
rs2569559	0.328107	0.86034	0.496842	0.497126	0.96276	0.49754	0.967898	0.496036	0.495942	0.495863	0.496107	0.49622	0.496389	0.495929	0.969527	

LD
≥0.9
[0.8,0.9)
[0.7,0.8)
[0.6,0.7)
[0.5,0.6)
[0.4,0.5)
[0.3,0.4)

Supplemental Figure 3. Myopia association for 16 variants in LD. The 16 variants in LD which all had associations with myopia are shown. They were subdivided into four categories (blue, green, purple, and red) based on level of LD, as shown in the table **(a)**. These variants have differing degrees of association with Hypercholesterolemia **(b)** or LDL-C **(c)** and myopia as plotted in the graphs. The individual variant with their associated categories (blue, green, purple, and red) dots are shown on the bottom of the graph corresponding to their nucleotide position in *LDLR*. The variants rs6511720 and rs2228671 are indicated by * and †, respectively. The full LD map containing these 16 variants is shown **(d)**.

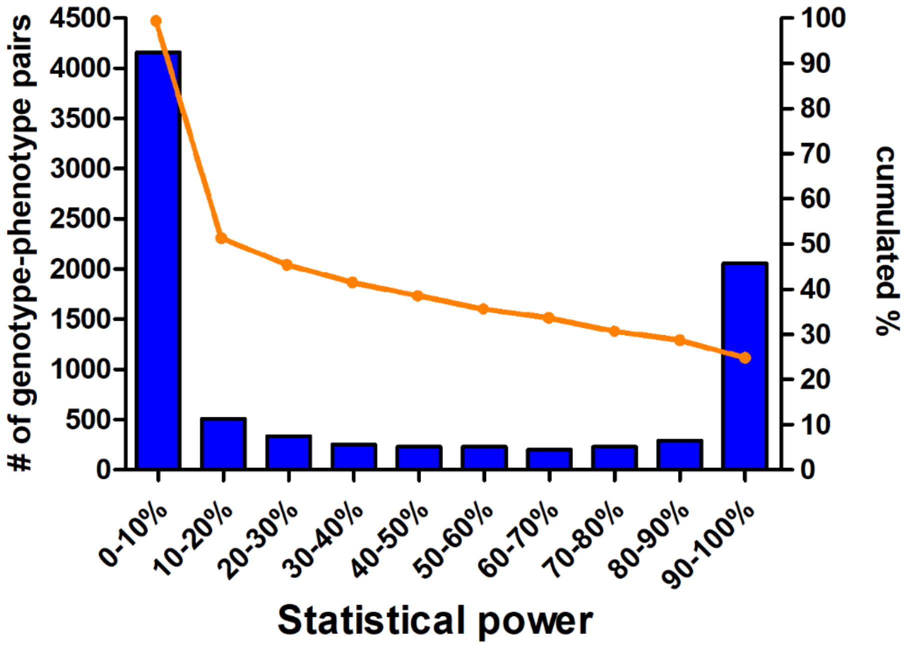

Supplementary Figure 4. Power for the 10 tested variants and 1,232 phecodes. The number of genotype-phenotype pairs for each 10% range of power is shown for European-ancestry individuals in the blue bars. Cumulated percentage of genotype-phenotype pairs for which had at least certain statistical power is shown in the orange line.

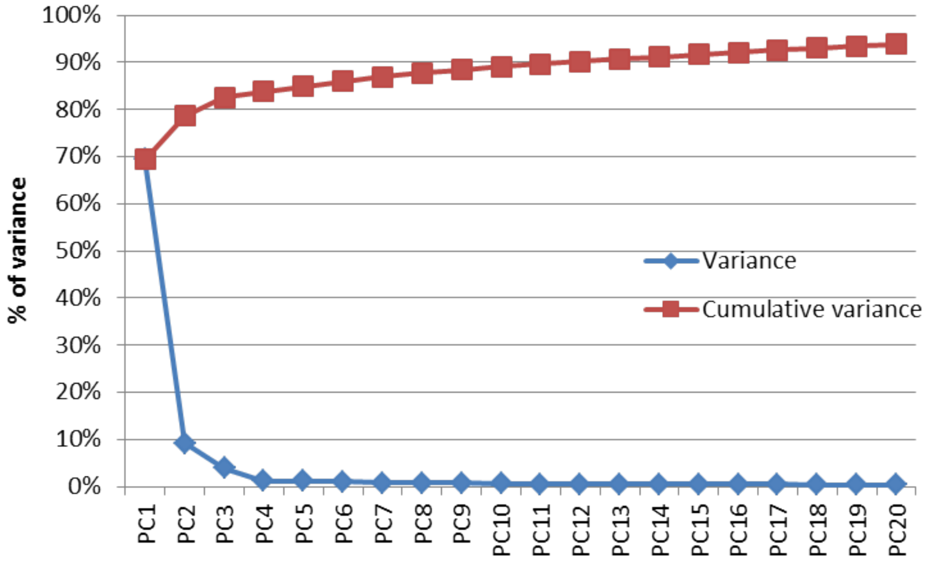

Supplementary Figure 5. Scree plot of principal component analysis.

Supplementary Table 1. Clinical sites comprising the eMERGE discovery cohort.

Cohort	Sample Size	Unrelated Adults With ICD-9 Codes	EA adults	AA adults	Non-EA, Non-AA adults
**Boston Children's Hospital**	1,023	18	11	5	2
**Columbia University Medical Center**	2,065	1,909	933	529	447
**Children's Hospital of Philadelphia**	10,465	68	35	30	3
**Cincinnati Children's Hospital Medical Center**	5,717	303	268	28	7
**Geisinger**	3,111	3,088	3,066	11	11
**Kaiser Permanente Washington Health Research Institute / University of Washington**	3,520	3,290	3,052	116	122
**Harvard Medical Center**	10,096	9,910	8,734	644	532
**Marshfield Clinic Research Foundation**	4,809	4,609	4,582	3	24
**Mayo Clinic**	10,261	9,123	9,026	28	69

**Mount Sinai Hospital**	6,255	6,124	973	4,510	641
**Northwestern University**	4,849	4,579	3,982	565	32
**Vanderbilt University**	21,814	21,137	17,038	3,807	292
**Total**	**83,985**	**64,589**	**51,700**	**10,276**	**2,182**

AA: African ancestry; EA: European ancestry

Supplementary Table 2. Variants that passed quality control filters for EA and AA in the secondary analysis.

Gene	Chr	Position	rsID	Ref	Alt	Annotation	MAF EA, %	MAF AA, %
PCSK9	1	55505668	rs11583680	T	C	missense	13.4	3.9
		55523802	rs28362261	G	A	missense		1.7
		55524237	rs562556	G	A	missense	17.1	2.1
		55529187	rs505151	G	A	missense	3.8	24.6
APOB	2	21224843	rs12713450	A	G	missense		11.0
		21224853	rs1801695	T	C	missense	3.4	
		21225281	rs1042034	T	C	missense	21.0	15.2
		21225485	rs1801702	G	C	missense		10.2
		21225597	rs61743299	T	A	missense		4.8
		21228827	rs1801701	T	C	missense	9.1	2.4
		21229905	rs12720854	C	T	missense		1.7

		21232803	rs584542	T	C	missense		4.9
		21232845	rs12713681	G	C	missense		2.1
		21233999	rs1801699	C	T	missense	1.9	
		21238007	rs61736761	T	G	missense		8.2
		21249716	rs12691202	T	C	missense	3.7	
		21250914	rs679899	A	G	missense		18.6
		21255355	rs12714225	G	A	missense		2.3
		21263900	rs1367117	G	A	missense	31.6	1.3
LDLR	19	11222300	rs11669576	A	G	missense	4.6	16.8

AA: African ancestry; EA: European ancestry

Quality control filters: Imputation quality r^2 >0.4; MAF >1%; missense mutation; no LCL-C association (EA threshold of 1.0×10^{-8}, AA threshold of 9.4×10^{-5}); LD r^2 <0.3

Supplementary Table 3. Phecodes[1] specific to diabetes (n=19).

Phecode	Description	Phecode	Description
250	Diabetes mellitus	250.24	Type 2 diabetes with neurological manifestations
250.1	Type 1 diabetes	250.25	Diabetes type 2 with peripheral circulatory disorders
250.11	Type 1 diabetes with ketoacidosis	250.4	Abnormal glucose
250.12	Type 1 diabetes with renal manifestations	250.41	Impaired fasting glucose
250.13	Type 1 diabetes with ophthalmic manifestations	250.42	Other abnormal glucose
250.14	Type 1 diabetes with neurological manifestations	250.6	Polyneuropathy in diabetes
250.15	Diabetes type 1 with peripheral circulatory disorders	250.7	Diabetic retinopathy
250.2	Type 2 diabetes	251.1	Hypoglycemia
250.22	Type 2 diabetes with renal manifestations	649.1	Diabetes or abnormal glucose tolerance complicating pregnancy
250.23	Type 2 diabetes with ophthalmic manifestations		

[1]**Post-quality control**

Supplementary Table 4. Phecodes[1] specific to neurocognitive disorders (n=85).

Phecode	Description	Phecode	Description
53.1	Herpes zoster with nervous system complications	335	Multiple sclerosis
191.1	Cancer of brain and nervous system	337.1	Peripheral autonomic neuropathy
191.11	Cancer of brain	345	Epilepsy, recurrent seizures, convulsions
225	Benign neoplasm of brain and other parts of nervous system	345.1	Epilepsy
225.1	Benign neoplasm of brain, cranial nerves, meninges	345.12	Partial epilepsy
253	Disorders of the pituitary gland and its hypothalamic control	345.3	Convulsions
253.7	Other disorders of neurohypophysis	348	Other conditions of brain
290	Delirium dementia and amnestic and other cognitive disorders	348.8	Encephalopathy, not elsewhere classified
290.1	Dementias	350	Abnormal movement
290.11	Alzheimer's disease	350.1	Abnormal involuntary movements

290.12	Dementia with cerebral degenerations	350.2	Abnormality of gait
290.2	Delirium due to conditions classified elsewhere	350.3	Lack of coordination
290.3	Other persistent mental disorders due to conditions classified elsewhere	351	Other peripheral nerve disorders
291.8	Alteration of consciousness	353	Nerve root and plexus disorders
292	Neurological disorders	353.1	Nerve plexus lesions
292.2	Mild cognitive impairment	353.2	Nerve root lesions
292.3	Memory loss	358	Myoneural disorders
292.4	Altered mental status	362.21	Macular degeneration, dry
295	Schizophrenia and other psychotic disorders	362.22	Macular degeneration, wet
295.1	Schizophrenia	362.23	Cystoid macular degeneration of retina
295.3	Psychosis	362.29	Macular degeneration (senile) of retina NOS
296	Mood disorders	362.6	Peripheral retinal degenerations
296.2	Depression	367.1	Myopia
296.22	Major depressive disorder	368	Visual disturbances

297	Suicidal ideation or attempt	368.2	Diplopia and disorders of binocular vision
297.1	Suicidal ideation	368.4	Visual field defects
297.2	Suicide or self-inflicted injury	368.9	Subjective visual disturbances
300.12	Agorophobia, social phobia, and panic disorder	368.91	Psychophysical visual disturbances
300.3	Obsessive-compulsive disorders	377.1	Optic atrophy
301.1	Schizoid personality disorder	377.3	Optic neuritis/neuropathy
303	Psychogenic and somatoform disorders	379.9	Pain, swelling or discharge of eye
303.3	Psychogenic disorder	386.2	Peripheral or central vertigo
303.4	Somatoform disorder	386.9	Dizziness and giddiness (Light-headedness and vertigo)
306	Other mental disorder	389.2	Conductive hearing loss
313.1	Attention deficit hyperactivity disorder	749	Congenital anomalies of face and neck
315	Develomental delays and disorders	752	Nervous system congenital anomalies
323	Encephalitis	764	Sciatica
323.8	Encephalitis, non-infectious	766	Neuralgia, neuritis, and radiculitis NOS

327	Sleep disorders	781	Symptoms involving nervous and musculoskeletal systems
327.6	Circadian rhythm sleep disorder	781.2	Abnormal posture
327.71	Restless legs syndrome	855	Complication of nervous system device, implant, and graft
332	Parkinson's disease	907	Injuries to the nervous system
334	Degenerative disease of the spinal cord		

[1]**Post-quality control**

Supplementary Table 5. Phecodes[1] pertinent to cataracts (n=6).

Phecode	Description
366	Cataract
366.1	Nonsenile Cataract
366.2	Senile cataract
366.3	Traumatic cataract
753.1	Congenital cataract and lens anomalies
368.4	Visual field defects

[1]Post-quality control
